# MLN51 Stimulates the RNA-Helicase Activity of eIF4AIII

**DOI:** 10.1371/journal.pone.0000303

**Published:** 2007-03-21

**Authors:** Christian G. Noble, Haiwei Song

**Affiliations:** Laboratory of Macromolecular Structure, Institute of Molecular and Cell Biology, Singapore, Singapore; Victor Chang Cardiac Research Institute, Australia

## Abstract

The core of the exon-junction complex consists of Y14, Magoh, MLN51 and eIF4AIII, a DEAD-box RNA helicase. MLN51 stimulates the ATPase activity of eIF4AIII, whilst the Y14-Magoh complex inhibits it. We show that the MLN51-dependent stimulation increases both the affinity of eIF4AIII for ATP and the rate of enzyme turnover; the *K*_M_ is decreased by an order of magnitude and *k*_cat_ increases 30 fold. Y14-Magoh do inhibit the MLN51-stimulated ATPase activity, but not back to background levels. The ATP-bound form of the eIF4AIII-MLN51 complex has a 100-fold higher affinity for RNA than the unbound form and ATP hydrolysis reduces this affinity. MLN51 stimulates the RNA-helicase activity of eIF4AIII, suggesting that this activity may be functionally important.

## Introduction

The processes of transcription, pre-mRNA processing, mRNA export, translation and degradation are all interconnected. For example, during splicing, an exon-junction complex (EJC) is deposited at a defined location ∼20–24 nucleotides upstream of exon-exon junctions in a sequence-independent manner [1]. The EJC remains stably associated with spliced mRNAs after their export to the cytoplasm, until it is removed by the ribosome during the “pioneering” round of translation [2]. The EJC increases the efficiency of mRNA export to the cytoplasm and stimulates translation [3], [4]. In mammals, the EJC serves as a molecular marker indicating the location of a splicing event that can trigger degradation of mRNAs containing a premature termination codon in a process termed nonsense-mediated mRNA decay (NMD) [5], [6].

The EJC is a dynamic complex which changes its composition during mRNA export into the cytoplasm [3], [7]. It consists of a central core of eIF4AIII, MLN51 (also known as Barentsz), Y14 and Magoh [8], [9]. In the nucleus, the EJC contains additional proteins including the splicing factors SRm160 and RNPS1 and the export factors UAP56, REF/Aly and TAP:p15, as well as the NMD protein Upf3b. The splicing and export factors are lost during mRNA export to the cytoplasm where additional NMD proteins bind to the EJC [7].

eIF4AIII is the third member of the eIF4A DEAD-box RNA-helicase family. It is functionally distinct from eIF4A isoforms I and II [10], which are required for translation initiation. All three eIF4A proteins have RNA-stimulated ATPase and ATP-dependent RNA-helicase activities [10]. The function of these activities for eIF4AIII within the EJC is not fully understood and eIF4AIII has been suggested to act as an ‘RNA clamp’ bound to a specific site on mRNA [11]. MLN51 was identified as a protein overexpressed in breast cancer [12] and a region called the SELOR domain (residues 137-283; MLN51 SELOR) has been shown to be sufficient for the interaction with eIF4AIII and RNA [13]. Y14 and Magoh form a stable complex through the binding of the RNA-binding domain (RBD) of Y14 to Magoh instead of RNA, suggesting that these proteins do not bind RNA in the context of the binary complex [14]–[16].

Previous biochemical studies have shown that MLN51 SELOR stimulates the RNA-dependent ATPase activity of eIF4AIII, whilst the Y14-Magoh complex inhibits this stimulation [9]. The inhibition of ATPase activity by Y14-Magoh increases the RNA-binding affinity of the EJC [9]. Recently, the crystal structure of the core EJC containing eIF4AIII, MLN51 SELOR, Magoh, Y14, AMPPNP and a short poly(U) RNA revealed that the two RecA-like domains of eIF4AIII enclose the ATP analogue and provide the binding site for the poly(U) mRNA mimic. MLN51 SELOR binds across both domains of eIF4AIII and Magoh-Y14 appears to inhibit ATP hydrolysis by locking the RNA-and ATP-bound state of eIF4AIII [17], [18].

In this study, we have further characterized RNA binding and the ATPase activity of the core components of the EJC. We show that MLN51 SELOR decreases the *K*_M,ATP_ of eIF4AIII by an order of magnitude and increases the turnover (*k*_cat_) 30 fold. The RNA-binding affinity of a complex of MLN51 SELOR and eIF4AIII is increased about 2-fold in the presence of ATP, but about 100-fold if Y14 and Magoh are also present. Also we show that the helicase activity of eIF4AIII is stimulated by MLN51, demonstrating that the eIF4AIII-MLN51 complex has RNA-helicase activity that may be functionally important.

## Results and Discussion

### eIF4AIII ATPase activity

**Figure 1:**
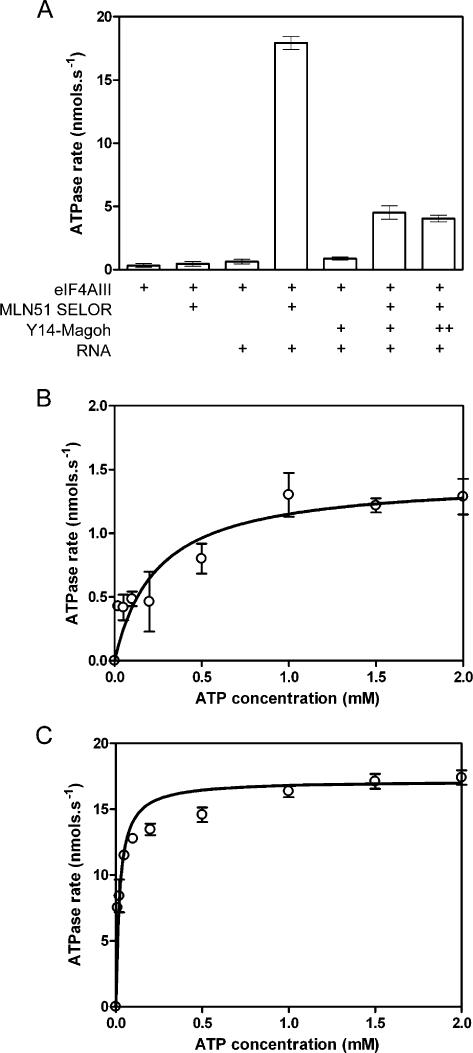
ATPase activity of eIF4AIII. (A) ATPase rates for 400 nM eIF4AIII in the presence or absence of 800 nM MLN51 SELOR, 800 nM Y14-Magoh and 200 µg.ml^−1^ yeast RNA. Increasing the Y14-Magoh concentration (lane 7; 3.2 µM) did not further inhibit the ATPase activity. RNA-dependent rates were measured at increasing ATP concentrations for 1 µM eIF4AIII alone (B) or for 400 nM eIF4AIII with 800 nM MLN51 SELOR (C) and the data were fitted to the Michaelis-Menten equation to determine values for *K*_M_ and *k*_cat_.

It has previously been shown that the RNA-dependent ATPase activity of eIF4AIII is stimulated by MLN51 SELOR and that this stimulated activity is inhibited by Y14-Magoh [9]. We have further characterized the ATPase activity of eIF4AIII using a more sensitive enzyme-linked ATPase assay. Recombinant eIF4AIII, the SELOR domain of MLN51 (MLN51 SELOR) and a complex of Magoh and N-terminally truncated Y14 (Y14-Magoh) were purified from *E. coli* and used in subsequent ATP-hydrolysis assays. eIF4AIII has a low background rate of ATP hydrolysis, which is stimulated approximately 2 fold in the presence of whole yeast RNA (Fig. 1A). This RNA-stimulated rate is further stimulated almost 30 fold by MLN51 SELOR. It is likely that this is due to the RNA-binding affinity of MLN51, bringing eIF4AIII into close proximity with RNA, since eIF4AIII has almost no affinity for RNA alone (see below) and MLN51 SELOR has no significant effect on the ATPase activity in the absence of RNA (Fig. 1A, lane 2). The Y14-Magoh complex does not inhibit the intrinsic RNA-stimulated activity of eIF4AIII but does inhibit the RNA-MLN51-stimulated ATPase activity. However even with a large excess of Y14-Magoh, the ATPase rate was not decreased back to the background level (Fig. 1A, lane 7), suggesting that this tetrameric complex may still slowly hydrolyze ATP. Consistent with this observation, in the crystal structure of the core EJC, all the residues at the ATP-hydrolysis site, including a catalytic water molecule, are in the correct position for catalysis [17].

Next we measured the RNA-stimulated ATPase rate at increasing concentrations of ATP in the presence or absence of MLN51 SELOR to determine the Michaelis-Menten constant (*K*_M_) and the rate of enzyme turnover (*k*_cat_) (Fig. 1B, C). In the absence of MLN51 SELOR the *K*_M_ is 240±100 µM whilst with MLN51 SELOR it is an order of magnitude lower at 22±6 µM, showing that MLN51 SELOR increases the affinity of eIF4AIII for ATP. The *k*_cat_ for eIF4AIII is 7 s^−1^, whilst with MLN51 SELOR as well it is 215 s^−1^, showing that MLN51 also increases the rate of enzyme turnover ∼30 fold. The higher error in the absence of MLN51 SELOR is due to a lower signal-to-noise ratio since the ATPase V_max_ is much lower. Nevertheless the values for *K*_M_ and *k*_cat_ for eIF4AIII alone are comparable to other DEAD-box RNA helicases [19]. Very few, if any, DEAD-box RNA helicases have been characterized in the presence of a binding partner that modulates the ATPase activity [19] and it is interesting to note that the values for *K*_M_ and *k*_cat_ for the eIF4AIII-MLN51 SELOR complex are respectively at least 3-and 20-fold higher than those published to date. Therefore, since these enzymes have relatively low rates of ATP hydrolysis alone, many other helicases may have binding partners that stimulate their activities.

### RNA binding

**Figure 2:**
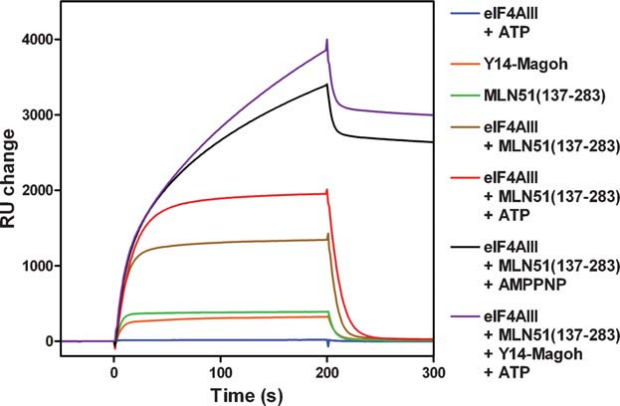
RNA binding by the core of the exon-junction complex. Binding to a biotinylated 35-mer RNA oligonucleotide was measured using surface plasmon resonance. 160 nM protein samples were injected across the chip surface and the interactions measured. The injections started at time zero and finished at 200 s. The sensorgrams are indicated in the key.

MLN51 SELOR has RNA-binding affinity [13], whilst the Y14-Magoh complex probably doesn't [14]–[16]. It is unclear whether eIF4AIII alone has affinity for RNA and whether the interaction is ATP-dependent. It has been reported that Y14, Magoh, MLN51, eIF4AIII, and ATP are all required for a stable RNA interaction [9]. To further characterize the EJC interaction with RNA, we have measured RNA binding using surface plasmon resonance. A 35-mer single-stranded RNA oligonucleotide was attached to the chip surface via a biotin group at the 5′ end, then protein samples were injected across the chip surface to see if they interacted (Fig. 2). The injections started at time zero and stopped at 200 s. No RNA binding was detected for eIF4AIII with or without a non-hydrolysable ATP analogue (AMPPNP) or ATP (Fig. 2, blue sensorgram). MLN51 SELOR interacted weakly with RNA (Fig. 2, green sensorgram) and the amount of protein bound to RNA increased when eIF4AIII was also added (Fig. 2, gold sensorgram), indicating that these proteins form a complex capable of interacting with RNA. The binding kinetics for MLN51 SELOR or eIF4AIII-MLN51 SELOR show rapid on and off rates and titrations were globally fitted to 1:1 binding models, both giving an equilibrium dissociation constant (*K*_d_) of ∼1 µM. This result shows that in the absence of ATP, MLN51 SELOR alone or complexed with eIF4AIII interacts with RNA weakly. When ATP was also added, the amount of eIF4AIII-MLN51 SELOR bound to the RNA increased ∼50% (Fig. 2, red sensorgram), but the kinetics remained similar with rapid on and off rates. Fitting the data to a 1:1 binding model gave a *K*_d_ of ∼0.5 µM. Since the binding on and off rates remain similar, and MLN51 stimulates the ATPase activity of eIF4AIII, these results suggest that ATP hydrolysis reduces the affinity of the eIF4AIII-MLN51 SELOR complex for RNA. Therefore, the ATP-bound form of the eIF4AIII-MLN51 SELOR complex may have a higher affinity for RNA than the ADP-bound or apo form. Consistent with this view, the crystal structure of the eIF4AIII-MLN51 complex in the absence of ATP, showed that eIF4AIII adopts an open conformation in which the RNA-binding site is disrupted [17].

We also examined the RNA interactions for eIF4AIII-MLN51 SELOR-AMPPNP and eIF4AIII-MLN51 SELOR-Y14-Magoh-ATP (Fig. 2, black and purple sensorgrams respectively). Both of these complexes have a similar association rate for RNA, but a very much slower dissociation rate than that seen for the eIF4AIII-MLN51 SELOR complex in the absence of a nucleotide, showing that RNA binding to these complexes is much tighter. (The small amount of rapid dissociation at ∼200 s is probably due to free protein not forming a tight complex with RNA.) Therefore the inhibition of the ATPase activity prevents the dissociation of the complex from RNA. When the data from a titration of these complexes are globally fitted to a 1:1 binding model, the *K*_d_ for RNA binding is ∼10 nM, an increase in affinity of 100 fold. The dissociation rate (*k*_d_) decreased by a similar amount, whilst the association rate remained unchanged, suggesting that ATP hydrolysis only affects dissociation from the RNA. Taken together, these data show that the ATP-bound eIF4AIII-MLN51 SELOR complex has a higher affinity for RNA than after the ATP is hydrolyzed. These results are similar to the related mammalian DEAD-box protein, eIF4AI [20]. The ATP-bound form of eIF4AI has a 40-fold higher affinity for single-stranded RNA than the ADP-bound form. The major difference from eIF4AIII is that eIF4AI shows this property alone, whilst eIF4AIII requires MLN51. Therefore high and lower affinity RNA binding may be a more common feature of DEAD-box RNA helicases, when accessory proteins are also taken into account.

It is still not clear whether the Y14-Magoh complex is involved in the interaction with RNA within the EJC. If it does bind to RNA it is probably not through the RBD since structures of the complex show that this domain forms an unusual protein-protein interaction with Magoh [14]–[16]. Therefore the Y14-Magoh complex was injected across the sensor chip to see if it interacted with the RNA oligonucleotide. A titratable response was detected (Fig. 2) which fitted to a *K*_d_ of ∼2 µM. The RNA-binding affinity and kinetics for Y14-Magoh are similar to MLN51 SELOR. These data suggest that the Y14-Magoh complex can weakly interact with RNA so it may stabilize the RNA interaction within the EJC. This is supported by UV-crosslinking experiments that suggest Y14-Magoh crosslinks to RNA in the context of the core EJC (data not shown).

### RNA-helicase activity

**Figure 3:**
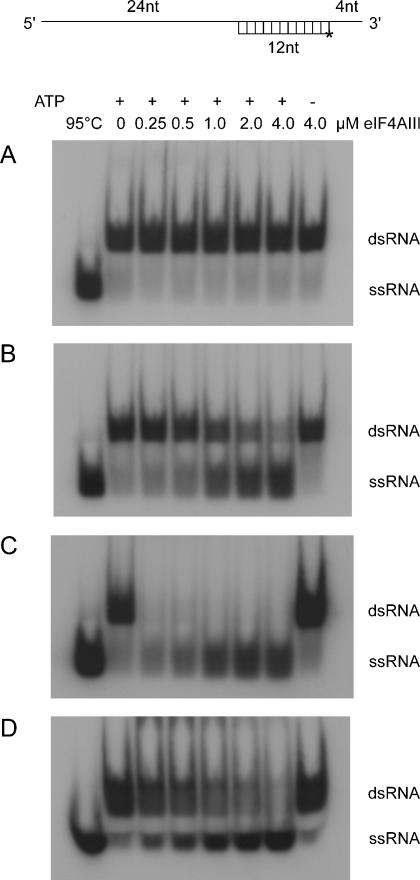
RNA-helicase activity of eIF4AIII. Helicase activity was assayed for 0.25 to 4 µM eIF4AIII with 10 nM ^32^P-labelled duplex RNA. Experiments were performed for (A) eIF4AIII alone, (B) with 5 µM MLN51 SELOR in lanes 2-8 or (C, D) with 5 µM MLN51 SELOR and Y14-Magoh in lanes 2-8. D is the same as C except the assays were terminated with a higher SDS concentration to disrupt the core EJC, affecting the resolution of the gel. The duplex was heated to 95°C (lane 1) as a marker for the migration of single-stranded RNA (ssRNA) and the input duplex double-stranded RNA (dsRNA), in the absence of additional protein, is shown in lane 2 top panel. A control assay in the absence of ATP was also performed (lane 8). A schematic illustration of the RNA substrate is shown at the top of the Figure.

eIF4AIII has been previously shown to have RNA-helicase activity alone [10], but the effects of the other components of the exon-junction complex were not tested. Although MLN51 stimulates the ATPase activity of eIF4AIII, it is not clear what effect it has on the RNA-helicase activity of eIF4AIII. Therefore we assayed the RNA-helicase activity of eIF4AIII alone and with MLN51 SELOR and/or Y14-Magoh (Fig. 3). The assays were performed using a 12-base-pair duplex with a 24-nucleotide 5′-single-stranded region and a 4-nucleotide 3′-single-stranded region (Fig. 3, top). The short strand was end labeled with ^32^P. In contrast to the previous finding that eIF4AIII has RNA-helicase activity, we did not detect any helicase activity for eIF4AIII alone despite using a similar RNA substrate [10]. However, when MLN51 SELOR was added to the reaction mixture, significant helicase activity was detected for eIF4AIII (Fig. 3B), and this activity was ATP-dependent. Surprisingly, RNA-helicase activity was also detected for eIF4AIII in the presence of both MLN51 SELOR and Y14-Magoh (Fig. 3C, D). Free single-stranded RNA is seen in the presence of Y14-Magoh (Fig. 3C), but the core EJC bound more tightly to RNA and a higher SDS concentration was required to fully disrupt the protein-RNA complex, affecting the resolution of the gel (Fig. 3D). Despite the presence of a large excess of Y14-Magoh over eIF4AIII in these assays we are unable to rule out the possibility that this activity is due to the eIF4AIII-MLN51 SELOR subcomplex.

Complexes of eIF4AIII and MLN51 and of Y14 and Magoh are considerably more stable than the quaternary complex containing all four proteins [8]. This is consistent with the structure of the core EJC, which shows that the dimer interfaces are largely hydrophobic, whereas the interface between eIF4AIII-MLN51 and Y14-Magoh is hydrophilic and contains a number of water molecules [17]. Therefore it is likely that the EJC assembles from these subcomplexes. Since the eIF4AIII-MLN51 complex has both ATPase and RNA-helicase activities, we suggest that this complex binds to pre-mRNA first and that it remodels the RNA, removing bound RNP. It is then joined by the more loosely-associated Y14-Magoh complex and perhaps additional components of the EJC. This complex may continue to hydrolyze ATP, perhaps remodeling loosely-associated RNP, before arresting in an ATP-inhibited state.

Once the mRNA is transported to the cytoplasm, the constituents of the EJC change. Many of the components of the EJC are lost during transport [3] and translation is required to remove Y14 from mRNA [2]. Therefore it is likely that the translating ribosome disrupts the interactions between the core EJC, activating the ATP hydrolysis and RNA helicase activities of eIF4AIII-MLN51, which may be required for RNP remodeling ahead of the ribosome. This could in turn stimulate the ‘pioneering’ round of translation. Splicing stimulates translation in mammalian cells [4] and this property is thought to be conferred through the positioning of the EJC which acts as a splicing-dependent marker. However it is not clear how components of the EJC, including Y14 and Magoh, actually stimulate translation. One possibility is that the eIF4AIII-dependent helicase activity increases translation efficiency by changing the local geometry of the RNA.

Further research is required to determine whether the core EJC containing eIF4AIII, MLN51, Y14 and Magoh has RNA-helicase activity. It may form a stable complex with RNA relatively slowly in the absence of other components of the EJC in order to prevent the EJC from ‘locking’ onto RNA non-specifically. If it requires interactions with other components of the EJC to load it onto RNA in vivo this would ensure that the complex only binds tightly to a specific location on the RNA, 20–24 nucleotides upstream of the exon-exon junction.

## Materials and Methods

### Protein expression and purification

The DNA sequence coding for Y14 (50–174) and full-length Magoh were isolated by PCR amplification from human cDNAs and cloned into pACYCDuet-1 (Novagen). Magoh was fused to an N-terminal hexa-histidine sequence using the EcoRI site and inserted 5′ to the native Y14 sequence (residues 50-174). eIF4AIII was isolated by the same method and cloned into pETDuet-1 (Novagen) and fused to the N-terminal hexa-histidine sequence using the BamHI site. The Y14-Magoh complex and eIF4AIII were expressed in the *E. coli* strain BL21 (DE3) and purified from clarified crude cell extracts by immobilized metal-ion-affinity, ion-exchange and gel-filtration chromatography. MLN51 (137–283) was isolated by PCR amplification, cloned into the expression vector pGEX6P-1 and expressed as a GST fusion in the *E. coli* strain BL21 (DE3). It was cleaved from a glutathione-affinity column using PreScission protease (Amersham Biosciences), followed by gel-filtration chromatography. The nucleotide sequence of each expression clone was verified by automated DNA sequencing. Protein concentrations were determined from the absorbance at 280 nm using molar extinction coefficients derived by summing the contributions from tyrosine and tryptophan residues.

### RNA oligonucleotides

The RNA oligonucleotides were purchased from Curevac. The oligonucleotide used for surface plasmon resonance experiments was 5′ to 3′: Biotin-AUGUCAUUCGAGUACAGUCUGUUCAGCUAGUCUCC. The oligonucleotides used in the helicase assays were similar to those used previously [10] and were 5′ to 3′: GGGGAGA(A_4_C)_3_UAGCACCGUAAAGCACGC and GCUUUACGGUGC.

Duplex RNA for RNA-helicase assays was formed by annealing 300 pmoles of labeled 12mer RNA with 400 pmoles of cold 40mer RNA in 10 mM Tris-HCl pH 8.0, 1 mM EDTA and 100 mM NaCl, by heating at 95°C and cooling slowly to room temperature.

### ATPase assays

ATPase assays were performed using an enzyme-linked assay consisting of lactate dehydrogenase and pyruvate kinase to link ATP hydrolysis to the oxidation of NADH which causes a decrease in the absorbance at 340 nm. The absorbance was converted into rate (nmols.s^−1^) using the extinction co-efficient for NADH of 6300 M^−1^.cm^−1^. Assays (200 µl) containing 2 mM ATP, 50 mM Tris-HCl pH 7.5, 50 mM NaCl, 5 mM DTT, 5 mM MgCl_2_, 1 mM phosphoenol pyruvate, 0.4 mM NADH, 1% (v/v) PK/LDH (Sigma) and 200 µg.ml^−1^ yeast RNA were measured at 25°C in a 96-well plate using a Benchmark Plus spectrophotometer (BioRad). eIF4AIII and MLN51 SELOR or Y14-Magoh were included at 400 and 800 nM respectively. For *K*_M_ measurements the ATP concentration was varied from 0.01 to 2 mM and the data were fitted to the Michaelis-Menten equation. For eIF4AIII alone the protein concentration was increased to 1 µM.

### Surface plasmon resonance

The 5′-biotinylated ribo-oligonucleotide was attached to a streptavidin-coated sensor chip (Biacore) as described previously [21]. Typically 100 µl of 10–160 nM protein sample was injected in 40 mM Tris-HCl pH 7.8, 50 mM NaCl, 4 mM DTT, 5 mM MgCl_2_, and 0.002% Tween 20 across the chip at 30 µl.min^−1^. Experiments were performed on a Biacore 2000 instrument. The data were fitted to a 1:1 binding model using BIAevaluation 3.2.

### RNA labeling and helicase assays

1 nmole of the 12mer ribo-oligonucleotide was end labeled using γ-^32^P-ATP and T4 polynucleotide kinase with standard protocols. The labeled RNA was purified from unincorporated ATP using G-25 Microspin columns (Amersham Biosciences) and then ethanol precipitated.

Helicase assays (20 µl) containing 10 nM duplex RNA and 0.25 to 4 µM eIF4AIII were performed in 40 mM Tris-HCl pH 7.8, 50 mM NaCl, 2 mM MgCl_2_, 3 mM DTT and 2 mM ATP and incubated at 37°C for 30 mins. 500 nM unlabelled 12mer RNA was also included in the reaction so that any free 40mer RNA that was generated was annealed to this excess of cold RNA. Reactions were terminated by adding 5 µl of 2% SDS, 50 mM EDTA, 50% glycerol and 0.01% bromophenol blue, then analyzed by electrophoresis at 12 mA for 45 minutes on native 12% acrylamide-TAE gels. The labeled components were visualized by autoradiography. Where indicated MLN51 SELOR and/or Y14-Magoh were included at 5 µM and reactions were terminated with 5 µl of 4% SDS, 50 mM EDTA, 50% glycerol and 0.01% bromophenol blue.

## References

[1] Le Hir H, Izaurralde E, Maquat LE, Moore MJ (2000). The spliceosome deposits multiple proteins 20-24 nucleotides upstream of mRNA exon-exon junctions.. EMBO J.

[2] Dostie J, Dreyfuss G (2002). Translation is required to remove Y14 from mRNAs in the cytoplasm.. Curr Biol.

[3] Le Hir H, Gatfield D, Izaurralde E, Moore MJ (2001). The exon-exon junction complex provides a binding platform for factors involved in mRNA export and nonsense-mediated mRNA decay.. EMBO J.

[4] Nott A, Le Hir H, Moore MJ (2004). Splicing enhances translation in mammalian cells: an additional function of the exon-junction complex.. Genes Dev.

[5] Lejeune F, Maquat LE (2005). Mechanistic links between nonsense-mediated mRNA decay and pre-mRNA splicing in mammalian cells.. Curr Opin Cell Biol.

[6] Parker R, Song H (2004). The enzymes and control of eukaryotic mRNA turnover.. Nat Struct Mol Biol.

[7] Tange TO, Nott A, Moore MJ (2004). The ever-increasing complexities of the exon-junction complex.. Curr Opin Cell Biol.

[8] Tange TO, Shibuya T, Jurica MS, Moore MJ (2005). Biochemical analysis of the EJC reveals two new factors and a stable tetrameric protein core.. RNA.

[9] Ballut L, Marchadier B, Baguet A, Tomasetto C, Seraphin B (2005). The exon-junction core complex is locked onto RNA by inhibition of eIF4AIII ATPase activity.. Nat Struct Mol Biol.

[10] Li Q, Imataka H, Morino S, Rogers GW, Richter-Cook NJ (1999). Eukaryotic translation initiation factor 4AIII (eIF4AIII) is functionally distinct from eIF4AI and eIF4AII.. Mol Cell Biol.

[11] Shibuya T, Tange TO, Sonenberg N, Moore MJ (2004). eIF4AIII binds spliced mRNA in the exon-junction complex and is essential for nonsense-mediated decay.. Nat Struct Mol Biol.

[12] Degot S, Regnier CH, Wendling C, Chenard MP, Rio MC (2002). Metastatic Lymph Node 51, a novel nucleo-cytoplasmic protein overexpressed in breast cancer.. Oncogene.

[13] Degot S, Le Hir H, Alpy F, Kedinger V, Stoll I (2004). Association of the breast cancer protein MLN51 with the exon-junction complex via its speckle localizer and RNA-binding module.. J Biol Chem.

[14] Fribourg S, Gatfield D, Izaurralde E, Conti E (2003). A novel mode of RBD-protein recognition in the Y14-Mago complex.. Nat Struct Biol.

[15] Lau CK, Diem MD, Dreyfuss G, Van Duyne GD (2003). Structure of the Y14-Magoh core of the exon-junction complex.. Curr Biol.

[16] Shi H, Xu RM (2003). Crystal structure of the *Drosophila* Mago nashi-Y14 complex.. Genes Dev.

[17] Bono F, Ebert J, Lorentzen E, Conti E (2006). The crystal structure of the exon-junction complex reveals how it maintains a stable grip on mRNA.. Cell.

[18] Andersen CB, Ballut L, Johansen JS, Chamieh H, Nielsen KH (2006). Structure of the exon junction core complex with a trapped DEAD-box ATPase bound to RNA.. Science.

[19] Cordin O, Banroques J, Tanner NK, Linder P (2006). The DEAD-box protein family of RNA helicases.. Gene.

[20] Lorsch JR, Herschlag D (1998). The DEAD-box protein eIF4A. 1. A minimal kinetic and thermodynamic framework reveals coupled binding of RNA and nucleotide.. Biochemistry.

[21] Noble CG, Walker PA, Calder LJ, Taylor IA (2004). Rna14-Rna15 assembly mediates the RNA-binding capability of *Saccharomyces cerevisiae* cleavage factor IA.. Nucleic Acids Res.

